# Alcohol

**Published:** 1872-06

**Authors:** 


### HALL'S

### JOURNAL OF HEALTH.

VOL. XIX.—JUNE, 1872.—NO. VI.

### ALCOHOL,

Or spirits of wine, is one half vapor of ether, and one half vapor of water. It is a clear, transparent, colorless, inflammable, volatile liquid, having an agreeable, penetrating odor, and a strong, burning taste. Its name came from the Arabs, who called it *Alkohl;* it has never been frozen; at a hundred and sixty-six below zero it becomes a little sticky. If it is deprived of all its water it is called absolute alcohol. In this state it is a most powerful poison, destroying life in a few hours. It weakens and thins the blood. Its first effects are to stimulate the brain, to make one more lively; then to dull and deaden all the faculties. When swallowed, it is correctly said to "go to the brain;" the brain first feels its effects; why it does so, can no more be told than why tartar emetic first affects the stomach. Everything sweet contains alcohol. Fruits, grains, vegetables, all have more or less sugar; the alcohol is got out of them by mixing them with water, and warming the mixture; this water and warmth causes decay, rotting, decomposition, called fermentation, by which the original constituent parts are separated.

The alcohol first rises up as a fume or gas, which cannot be seen; if this gas is carried away through a pipe, surrounded by cold water or cold air, it condenses on the inside, in drops which run together, until a little stream is formed, coming out at the other end of the pipe in the form of alcohol, one of the most useful substances in nature, of incomparable value in the mechanic arts in innumerable directions.

It is alcohol which causes drunkenness, and whatever fluid has the most alcohol in it, makes a man drunk the soonest, and the soonest destroys soul, body, and estate.

The various "liquors" which are in use have their names given them according to the source from which the alcohol has been obtained.

Brandy is alcohol obtained from grapes.

Cider is alcohol obtained from apples.

Gin is alcohol flavored with the juniper berry, originally, but now formed by adding other ingredients to alcohol. British gin, for example, is made by adding oil of turpentine to common whiskey, which in its turn is alcohol derived from Indian corn, barley, and other grains.

Beer, ale, and porter, all have alcohol in them; the more alcohol, the more valued, — lager beer being the most highly prized; "lager" meaning stronger, because, being longer kept, there is more alcohol in it.

Wine of every kind now used as an article of merchandise, or as a beverage, contains more or less alcohol; a wine destitute of alcohol is no wine at all, and would never prove a profitable investment.

